# Composition and Antimicrobial Activity of *Euphrasia rostkoviana* Hayne Essential Oil

**DOI:** 10.1155/2015/734101

**Published:** 2015-04-27

**Authors:** Pavel Novy, Hana Davidova, Cecilia Suqued Serrano-Rojero, Johana Rondevaldova, Josef Pulkrabek, Ladislav Kokoska

**Affiliations:** ^1^Department of Quality of Agricultural Products, Faculty of Agrobiology, Food and Natural Resources, Czech University of Life Sciences Prague, Kamycka 129, 165 21 Prague, Czech Republic; ^2^Department of Crop Sciences and Agroforestry, Faculty of Tropical AgriSciences, Czech University of Life Sciences Prague, Kamycka 129, 165 21 Prague, Czech Republic; ^3^Department of Crop Production, Faculty of Agrobiology, Food and Natural Resources, Czech University of Life Sciences Prague, Kamycka 129, 165 21 Prague, Czech Republic

## Abstract

Eyebright, *Euphrasia rostkoviana* Hayne (Scrophulariaceae), is a medicinal plant traditionally used in Europe for the treatment of various health disorders, especially as eyewash to treat eye ailments such as conjunctivitis and blepharitis that can be associated with bacterial infections. Some *Euphrasia* species have been previously reported to contain essential oil. However, the composition and bioactivity of *E. rostkoviana* oil are unknown. Therefore, in this study, we investigated the chemical composition and antimicrobial activity of the eyebright essential oil against some organisms associated with eye infections: *Enterococcus faecalis*, *Escherichia coli*, *Klebsiella pneumoniae*, *Staphylococcus aureus*, *S. epidermidis*, *Pseudomonas aeruginosa*, and *Candida albicans*. GC-MS analysis revealed more than 70 constituents, with n-hexadecanoic acid (18.47%) as the main constituent followed by thymol (7.97%), myristic acid (4.71%), linalool (4.65%), and anethole (4.09%). The essential oil showed antimicrobial effect against all organisms tested with the exception of *P. aeruginosa*. The best activity was observed against all Gram-positive bacteria tested with the minimum inhibitory concentrations of 512 *µ*g/mL. This is the first report on the chemical composition of *E. rostkoviana* essential oil and its antimicrobial activity.

## 1. Introduction

Eyebright,* Euphrasia rostkoviana* Hayne (Scrophulariaceae), has been used in Europe for centuries as a traditional medicine for treatment of various diseases. Decoctions and infusions of flowering aerial parts are used against dry cough, hoarseness, symptomatic treatment of cold, earache, and headache, hay fever, purulent skin lesion, or catarrhal diseases of the intestinal tract, but especially as eyewash to treat and prevent eye disorders such as conjunctivitis, blepharitis, eye fatigue, purulent ocular inflammation, and sties [1–3]. The use of eyebright tea has also been reported in ethnoveterinary medicine for cow eye infection treatment [4]. Despite centuries of the traditional use for the treatment of eye ailments, there has been only one prospective cohort trial carried out confirming the efficacy of* Euphrasia* eye drops in the treatment of conjunctivitis [5] and a single clinical study investigating the effect of local application of the eye drops on antibiotic consumption in neonates [6]. Moreover, until the recent reports on anticandidal [7] and antibacterial activity [8] of some* Euphrasia* extracts, the spectrum of antimicrobial action has been completely unknown.

The therapeutic effect of* E. rostkoviana* can be attributed mainly to its antioxidant, anti-inflammatory, and antimicrobial activity [2, 4, 8, 9]. Among the compounds previously identified in* E. rostkoviana* extracts [8–10], apigenin, luteolin, kaempferol, quercetin, caffeic acid, coumaric acid, and rosmarinic acid may be responsible for the antimicrobial action. Although the presence of essential oil (EO) in* E. officinalis* L. [11] and* E. stricta* Kunt [12] has previously been reported, the composition and bioactivity of the* E. rostkoviana* EO are unknown. Therefore, in this study, we investigated the chemical composition and antimicrobial activity of the eyebright EO against the panel of three Gram-positive bacteria (*Enterococcus faecalis*,* Staphylococcus aureus*, and* S. epidermidis*) and three Gram-negative bacteria (*Escherichia coli*,* Klebsiella pneumoniae*, and* Pseudomonas aeruginosa*), and one yeast (*Candida albicans*), organisms commonly associated with eye infections.

## 2. Material and Methods 

### 2.1. Chemicals and Plant Material

The authentic standards borneol, camphor, carvacrol, carvone, caryophyllene, p-cymene, estragole, eucalyptol, limonene, linalool, menthol, menthone, *β*-myrcene, *γ*-terpinene, and thymol for EO components identification as well as the control antibiotics ciprofloxacin and tioconazole were purchased from Sigma-Aldrich (Prague, Czech Republic). Hexane (Merck, Prague, Czech Republic), dimethyl sulfoxide (DMSO) (Lach-Ner, Neratovice, Czech Republic), and Tween 80 (Sigma-Aldrich, Prague, Czech Republic) were used as solvents. The plant material used for the EO distillation was purchased from commercial sources (F-DENTAL, Hodonín, Czech Republic). The EO was extracted by hydrodistillation using Clevenger type apparatus.

### 2.2. Chemical Analysis of the EO by Gas Chromatography-Mass Spectrometry (GC-MS)

The* E. rostkoviana* EO was analyzed by GC-MS using Agilent 7890A GC coupled to Agilent 5975C single-quadrupole mass detector equipped with a HP-5MS column (30 m × 0.25 mm, 0.25 *μ*m film) from Agilent (Santa Clara, CA, USA). Hexane was used as a solvent and the sample volume of 1 *μ*L was injected in split mode (ratio 20 : 1) into the injector heated to 250°C. The starting oven temperature was set at 60°C for 3 min, programmed to 230°C at a rate of 3°C/min, and then kept constant for 10 min. Helium was used as carrier gas with the flow rate of 1 mL/min. The MS analysis was carried out in full-scan mode and the electron ionization energy was set at 70 eV. The identification of individual components was based on the comparison of their mass spectra and relative retention indices with the National Institute of Standards and Technology Library (NIST, USA) and literature [13], as well as coinjection of authentic standard.

### 2.3. Bacterial Strains and Cultivation Media

The standard strains of three Gram-positive bacteria* Enterococcus faecalis* ATCC 29212,* Staphylococcus aureus* ATCC 29213, and* S. epidermidis* ATCC 12228, three Gram-negative bacteria* Escherichia coli* ATCC 25922,* Klebsiella pneumoniae* ATCC 700603, and* Pseudomonas aeruginosa* ATCC 27853, and one yeast* Candida albicans* ATCC 10231 were obtained from Oxoid (Basingstoke, United Kingdom). Cation adjusted Mueller-Hinton broth (MHB) and Sabouraud dextrose broth (SDB) were used as cultivation media for antibacterial and antifungal microdilution assay, respectively, and were equilibrated with Tris-buffered saline (Sigma-Aldrich, Prague, Czech Republic). Mueller-Hinton agar (MHA) and Sabouraud dextrose agar (SDA) were used for subsequent determination of bactericidal and fungicidal concentrations, respectively. All media were purchased from Oxoid (Basingstoke, United Kingdom).

### 2.4. Minimum Inhibitory Concentration (MIC) Determination

The MICs were determined using the* in vitro* broth microdilution method following the guidelines of Clinical and Laboratory Standards Institute (CLSI) [14, 15] modified according to the recommendations proposed for effective assessment of the anti-infective potential of natural products [16] using 96-well microtiter plates. Briefly, the EO was dissolved in DMSO with addition of Tween 80 and two-fold serial dilutions were prepared in MHB for bacteria and in SDB for the yeast whereas the concentrations tested ranged from 4 to 2048 *μ*g/mL. The inoculum was prepared from overnight cultures so that the initial CFU concentrations in the microplates were 5 × 10^5^ and 2 × 10^3^ CFU/mL for bacteria and yeast, respectively. The inoculated plates were examined after 24 h of incubation at 35°C and once more after 48 h in case of* C. albicans*. The microbial growth was measured spectrophotometrically by Multiscan Ascent Microplate Photometer (Thermo Fisher Scientific, Waltham, USA) at 405 nm. MICs were expressed as the lowest concentrations able to inhibit ≥ 80% of bacterial growth compared to the positive growth control. The experiments were performed in triplicate in three independent tests and median values were used for MICs calculation. Due to the recently reported possibility of EO volatile components' influence on the microbial growth in adjoining wells [17], positive growth control rows were inserted in between the EO dilution rows to detect eventual growth influence. The solvents used did not inhibit the bacterial growth at concentrations tested. Ciprofloxacin and tioconazole were used as reference antibiotics for bacteria and yeast, respectively.

### 2.5. Minimum Bactericidal Concentration (MBC) and Minimum Fungicidal Concentration(MFC) Determination

The aliquots of 20 *μ*L were transferred from each well without microbial growth to the MHA plates (SDA plates for* C. albicans*) after 24 h and 48 h of incubation of bacteria and yeast tested, respectively. The plates were then incubated for 24 h at 35°C. The MBC and MFC were evaluated as ≥99.9% decrease in CFU comparing to inoculum, all performed in triplicate in three independent tests.

## 3. Results and Discussion 

### 3.1. Chemical Characterization of Oils and Bioactive Fractions Constituents

**Figure 1:**
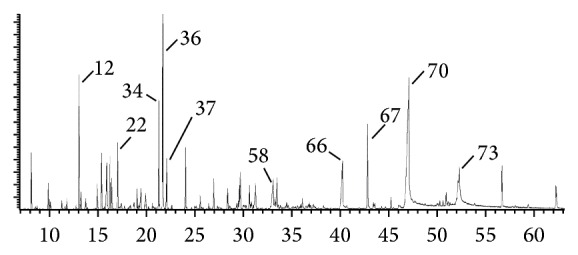
Typical chromatogram of* Euphrasia rostkoviana* essential oil. The main components are labeled according to the order of their retention times. (12) Linalool; (22) *α*-terpineol; (34) anethole; (36) thymol; (37) carvacrol; (58) lauric acid; (66) myristic acid; (67) hexahydrofarnesyl acetone; (70) palmitic acid; (73) linolenic acid.

The EO hydrodistillation by Clevenger-type apparatus yielded 0.02% (w/v) of yellowish-brown oil that tends to solidify at room temperature which is probably caused by high proportion of fatty acids (32.23% in total). GC-MS analysis of the EO revealed the presence of more than 70 constituents, with palmitic acid (18.47%) being the most abundant component followed by thymol (7.97%), myristic acid (4.71%), linalool (4.65%), anethole (4.09%), linolenic acid (3.81%), hexahydrofarnesyl acetone (3.16%), lauric acid (2.79%), *α*-terpineol (2.39%), and borneol (2.39%). The main compounds are shown also in the chromatogram (Figure 1) and the complete list of EO constituents is presented in Table 1.

The high content of fatty acids has previously been found in the* E. stricta* EO (25.7% in total) also with the highest proportion of palmitic acid (20.3%) and myristic acid (3.9%) [12]. However, there is no other compound present in significant amount that would indicate the relatedness of these two* Euphrasia* species.

### 3.2. Antimicrobial Activity

The* E. rostkoviana* EO showed activity against six out of seven organisms tested with MICs ranging from 512 to 2048 *μ*g/mL. The Gram-positive bacteria were more sensitive than the Gram-negative ones and the yeast whereas* P. aeruginosa* was the only organism that was not inhibited by the oil at the highest concentrations tested. The MICs, MBCs, and MFCs of the EO against all microorganisms tested are summarized in Table 2. The active concentrations are comparable to those previously reported for, for example, EOs of* Artemisia annua, Eucalyptus globulus*,* Mentha suaveolens*,* Myrtus communis*,* Ocimum basilicum*, or* Thymus vulgaris*, especially in the case of anticandidal activity [18–20]. The oil was also more effective than* E. rostkoviana* extracts tested by Teixeira and Silva [8] against* E. coli*,* E. faecalis*,* S. aureus*, and* S. epidermidis*. The MICs of the reference antibiotics against the bacteria and yeast susceptible to the* E. rostkoviana* EO were in accordance with the CLSI acceptable limits and previous reports, respectively [21–23].

Since the content of the main EO constituent palmitic acid does not exceed 20% and there are more than 10 other antimicrobially active compounds ranging from 1 to 8% it is difficult to suggest the main agents responsible for the* E. rostkoviana* EO antimicrobial effect. Palmitic acid has been previously identified as the major compound of fractions active against Gram-negative, but not Gram-positive, bacteria [24]. On the other hand, medium-chain saturated fatty acids and long-chain unsaturated fatty acids are known to inhibit especially Gram-positive bacteria [25]. Moreover, lauric acid exerts also activity against a number of fungi [26]. Thus the antimicrobial activity of the EO is probably due to a complex action of the antimicrobial fatty acids with the other well-known antimicrobial compounds identified in the EO such as anethole, borneol, camphor, carvacrol, linalool, menthol, *α*-terpineol, or thymol.

## 4. Conclusions

In conclusion, the chemical analysis revealed a number of antimicrobially active substances present in the* E. rostkoviana* EO and its antifungal and antibacterial activity against Gram-positive as well as Gram-negative bacteria was confirmed. To the best of our knowledge, this is the first report on the composition and antimicrobial activity of* E. rostkoviana* EO.

## Figures and Tables

**Table 1:** Chemical composition of *Euphrasia rostkoviana* Hayne essential oil.

Peak number	Component	RI	Area (%)^∗^	ID	Peak number	Component	RI	Area (%)	ID
1	1-Hexanol^d^	—	0.10	a	41	Damascenone	1385	0.56	a, b
2	1-Octen-3-ol	981	1.82	a, b	42	Methyl eugenol	1406	0.23	a, b
3	*β*-Myrcene	992	0.14	a, b, c	43	Caryophyllene	1419	1.28	a, b, c
4	3-Octanol	996	0.13	a, b	44	Geranyl acetone	1455	0.89	a, b
5	p-Cymene	1027	0.81	a, b, c	45	Trans-*β*-farnesene	1460	0.13	a, b
6	Limonene	1032	0.34	a, b, c	46	Alloaromadendrene	1462	0.12	a, b
7	Eucalyptol	1034	0.25	a, b, c	47	*γ*-Muurolene	1478	0.25	a, b
8	*γ*-Terpinene	1062	0.34	a, b, c	48	Germacrene D	1482	0.31	a, b
9	Sabinene hydrate	1070	0.14	a, b	49	Curcumene	1484	1.21	a, b
10	1-Octanol	1074	0.40	a, b	50	Trans-*β*-ionone	1487	1.53	a, b
11	3,5-Octadienone^d^	1094	0.16	a, b	51	Valencene	1493	0.13	a, b
12	Linalool	1101	4.65	a, b, c	52	*α*-Selinene^d^	1495	0.17	a, b
13	*α*-Thujone	1106	0.88	a, b	53	Epizonarene^d^	1498	0.26	a, b
14	*β*-Thujone	1118	0.48	a, b	54	*α*-Muurolene	1500	0.18	a, b
15	Camphor	1146	1.00	a, b, c	55	*β*-Bisabolene	1510	0.94	a, b
16	Menthone	1156	1.98	a, b, c	56	*γ*-Cadinene	1515	0.40	a, b
17	2-Nonenal, (E)-	1163	0.10	a, b	57	Nerolidol^d^	1566	0.10	a, b
18	Borneol	1168	2.39	a, b, c	58	Lauric acid	1574	2.79	a, b
19	(+/−)Lavandulol	1170	0.16	a, b	59	Spathulenol	1578	0.61	a, b
20	Menthol	1175	2.02	a, b, c	60	Caryophyllene oxide	1583	1.47	a, b
21	4-Terpineol	1179	1.13	a, b	61	Pseudoionone^d^	1587	0.18	a, b
22	*α*-Terpineol	1191	2.39	a, b	62	Humulene epoxide II	1609	0.25	a, b
23	Estragole	1199	0.34	a, b, c	63	Longifolenaldehyde^d^	1613	0.20	a, b
24	Decanal	1207	0.15	a, b	64	*τ*-Cadinol	1643	0.18	a, b
25	*β*-Cyclocitral^d^	1222	0.21	a, b	65	*β*-Eudesmol	1651	0.13	a, b
26	Thymol methyl ether	1238	0.75	a, b	66	Myristic acid	1771	4.71	a, b
27	Cumin aldehyde	1242	0.23	a, b	67	Hexahydrofarnesyl acetone	1847	3.16	a, b
28	Neral	1244	0.14	a, b	68	Pentadecanoic acid	1865	0.28	a, b
29	Carvone	1246	1.28	a, b, c	69	Farnesyl acetone	1919	0.50	a, b
30	Piperitone	1256	0.18	a, b	70	Palmitic acid	1977	18.47	a, b
31	Geraniol	1258	0.79	a, b	71	Phytol^d^	2114	0.12	a, b
32	Trans-2-decenal^d^	1264	0.11	a, b	72	Linoleic acid	2143	1.90	a, b
33	Geranial	1273	0.15	a, b	73	Linolenic acid	2148	3.81	a, b
34	Anethole	1287	4.09	a, b	74	Tricosane	2300	1.79	a, b
35	Safrole	1289	0.21	a, b	75	Tetracosane	2400	0.16	a, b
36	Thymol	1295	7.97	a, b, c	76	Pentacosane	2500	1.36	a, b
37	Carvacrol	1304	1.96	a, b, c					
38	(E,E)-2,4-Decadienal	1318	0.21	a, b		Total identified		**98.91**	
39	Capric acid	1374	0.26	a, b					
40	*α*-Copaene	1377	0.15	a, b					

R: retention indices relative to n-alkanes on HP-5MS capillary column (30 m × 0.25 mm, 0.25 *μ*m); ^∗^peak area relative to total peak area in %; ID: identification method; a: identification based on mass spectra matching; b: identification based on retention index; c: identification based on coinjection of authentic sample; d: tentative identification.

**Table 2:** The inhibitory and cidal concentrations of *E. rostkoviana* essential oil.

Microorganism	*Euphrasia rostkoviana* EO					CIP	TIO
	MIC (*µ*g/mL)		IC_50_ (*µ*g/mL)		MBC/MFC (*µ*g/mL)	MIC (*µ*g/mL)	MIC (*µ*g/mL)
	24 h	48 h^∗^	24 h	48 h	24 h	24 h	48 h
*Enterococcus faecalis *	512	—	128	—	1024	0.5	—
*Staphylococcus aureus *	512	—	128	—	>2048	0.5	—
*Staphylococcus epidermidis *	512	—	256	—	>2048	0.25	—
*Klebsiella pneumoniae *	2048	—	1024	—	>2048	0.125	—
*Escherichia coli *	2048	—	1024	—	>2048	0.015	—
*Pseudomonas aeruginosa *	>2048	—	>2048	—	>2048	0.125	—
*Candida albicans *	128	1024	128	1024	2048	—	0.063

^∗^The growth inhibition was measured after 24 h and 48 h of incubation in case of *C. albicans*; EO: essential oil; CIP: ciprofloxacin; TIO: tioconazole; MIC: minimum inhibitory concentration; IC50: inhibitory concentration causing ≥50% of bacterial growth; MBC: minimum bactericidal concentration; MFC: minimum fungicidal concentration.
